# Compliance, adherence and effectiveness of a community-based pre-operative exercise programme: a pilot study

**DOI:** 10.1186/s13741-019-0126-y

**Published:** 2019-12-02

**Authors:** Lisa Loughney, Ronan Cahill, Kiaran O’Malley, Noel McCaffrey, Brona Furlong

**Affiliations:** 1 MedEx Wellness, School of Health and Human Performance, Dublin City University, Glasnevin, Dublin, Ireland; 2 Department of Colorectal Surgery, Mater Misericordiae University Hospital, Dublin, Ireland; 3 Section of Surgery and Surgical Science, University College Dublin, Dublin, Ireland; 4 Department of Urology Surgery, Mater Misericordiae University Hospital, Dublin, Ireland

**Keywords:** Surgical-oncology, Prostate cancer, Colorectal cancer, Surgery, Community, Pre-operative Exercise training

## Abstract

**Background:**

Pre-operative exercise training improves HR components of fitness and HRQoL following hospital-based programmes.

**Objective:**

To assess compliance and adherence of a pragmatic community-based preoperative exercise programme and its effect on health-related (HR) components of fitness and health-related quality of life (HRQoL).

**Methods:**

Thirty-two surgical oncological participants (15 prostate cancer and 17 colorectal cancer (CRC)) were recruited and assessed to measure HR components of fitness (strength and functional exercise capacity) and HRQoL. An exercise programme was prescribed in the time available prior to surgery with repeat assessments pre-operatively.

**Results:**

Twenty-four participants (14 prostate cancer and 10 CRC) completed the full study (75% compliance). Exercise training was delivered over a median interquartile range (IQR) of 4 (3-4) weeks and 2 (1–3) weeks for the prostate cancer and CRC participants, with > 80% adherence. From baseline to post-exercise intervention, there were significant improvements in lower body strength in the prostate cancer group (*p* = 0.045), the CRC group (*p* = 0.001), and in both groups overall (*p* = .001). Additionally, there were statistically significant improvements in HRQoL: global health status for CRC group (*p* = 0.025) and for both groups overall (*p* = 0.023); emotional health subscale for the prostate cancer group (*p* = 0.048) and for both groups overall (*p* = 0.027); nausea/vomiting/pain subscale for the CRC group (*p* = 0.005) and for both groups overall (*p* = 0.030); and for health scale status for the prostate cancer group (*p* = 0.019) and for both groups overall (*p* = 0.006).

**Conclusion:**

This community-based pre-operative exercise programme showed acceptable compliance and adherence rates, and significantly increased upper and lower body strength and HRQoL. Pre-operative exercise training should be considered as early as possible in the surgical-oncology pathway and respected within patient scheduling.

## Background

Surgery is the mainstay treatment for people with newly diagnosed prostate and colorectal cancer (CRC) on a curative pathway. The level of risk associated with surgery has been described in a large European Surgical Outcome Study which showed that the mortality rate for patients undergoing inpatient non-cardiac surgery was higher than anticipated (Pearce and Grocott 2011). However, morbidity following major surgery is more common than mortality, impairs the recovery process post-operatively and is associated with long-term health implications (Moonesinghe et al. 2014). There is a strong evidence base showing that reduced pre-operative physical fitness levels are strongly associated with post-operative morbidity (Older et al. 1993; Hartley et al. 2012; Prentis et al. 2012; West et al. 2016; Thompson et al. 2011; Hennis et al. 2012; Snowden et al. 2010).

The importance of exercise in the cancer journey has been recently highlighted in a report by the Clinical Oncology Society of Australia with the clear recommendation that exercise should be embedded as part of standard practice in cancer care (Cormie et al. 2019). Pre-operative exercise training optimises physical fitness enabling an individual to maintain a better function during and after surgery. It has been shown to significantly improve fitness and health-related quality of life (HRQoL) (Mujovic et al. 2014; West et al. 2014; Barakat et al. 2014), however, much of this work has been reported following hospital-based (Mujovic et al. 2014; West et al. 2014; Barakat et al. 2014) and some home-based (Coats et al. 2013; Gillis et al. 2014) programmes. A recent report demonstrated that a supervised pre-operative exercise programme (as part of a multi-modal prehabilitation programme) showed significant improvements on functional capacity and muscle strength and had two times higher chances of returning to baseline fitness after surgery compared to the home-based exercise group (Awasthi et al. 2018).

Community-based exercise programmes are recommended interventions by the World Health Organization to promote physical activity for people living with an illness. They include a structured set of exercises designed for individuals with specific exercise needs and commonly involve a group of people with similar illnesses exercising under the supervision of a physiotherapist or a fitness instructor, with the goal of promoting and continuing regular exercise in the community (Salbach et al. 2014; Smith et al. 2011; Stuart et al. 2009). Community-based exercise programmes are attractive as they represent a more accessible, scalable, and sustainable alternative to hospital-based programmes and may reduce the burden on the healthcare system. Although few studies have explored community-based training in the pre-operative setting, the early data is encouraging and shows that they are feasible and effective (Rao et al. 2012; Singh et al. 2017).

The aim of this study was firstly to determine the compliance and adherence of a community-based pre-operative exercise training programme (delivered in a leisure centre) using a pragmatic approach (within whatever time interval was available before surgery) and secondly to investigate its effectiveness on selected health-related (HR) components of fitness and HRQoL for people with newly diagnosed prostate and CRC.

## Methods

### Study design

**Fig. 1:**
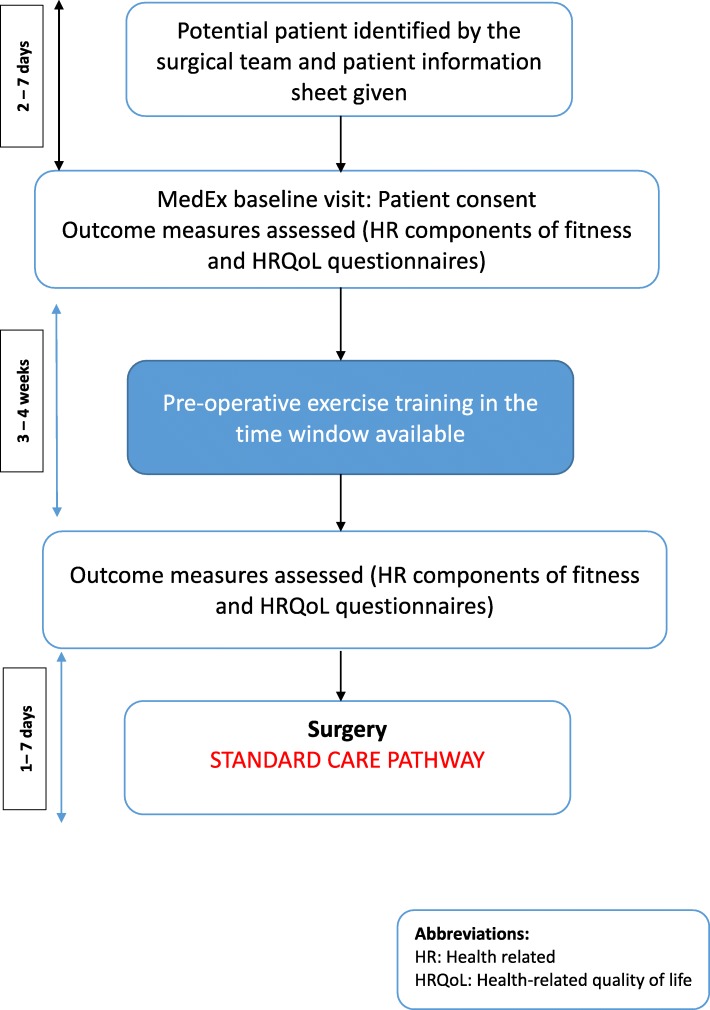
Study flow algorithm illustrating patient pathway

This pre-post-intervention pilot study investigated the compliance, adherence and effects of a community-based pre-operative exercise training programme on HR components of fitness and HRQoL. The study was approved by Dublin City University Research Ethics Committee (REC/2015/207) with patients referred from the Mater Misericordiae University Hospital (MMUH). The study flow diagram is presented in Fig. 1.

### Participants

Eligibility for inclusion at cancer diagnosis included the following: aged ≥ 18 years with a clinical diagnosis of prostate or CRC being scheduled for surgery (with or without neoadjuvant cancer treatment in the case of the latter). The exclusion criteria included contraindications to exercise including uncontrolled cardiovascular conditions, significant skeletal muscle, orthopaedic, neurological conditions, or cognitive decline, significant mental illness or intellectual disability that prevented participation in a physical training programme, as per physician discretion (ATS/ACCP 2003). From June 2016 to June 2018, eligible patients with a new prostate or CRC cancer diagnosis being scheduled for surgery were given a patient information leaflet by either their surgical consultant or clinical nurse specialist in the outpatient cancer clinic. Interested patients made contact or were contacted by the lead study clinical exercise physiologist (LL) (this was dependent on outpatient clinic, i.e. one consultant asked patients to contact the researcher and the other referred patients directly). Note: some patients referred from clinic lived in remote areas outside of Dublin therefore they were prescribed a home-programme not a centre-based programme. Written informed consent was provided at the baseline visit. Participant medical co-morbidity and medication list were obtained at this visit.

### Community-based exercise intervention

MedEx (now rebranded as ExWell Medical) is an established medically supervised chronic illness rehabilitation programme delivered in a leisure centre “(https://www.exwell.ie/)”. The exercise intervention for this study was delivered as part of the MedEx programme and involved attending the MedEx centre at 10 am, any week day, from Monday to Friday. Participants undertook exercise training as part of the MedEx cancer prepare service which is a peri-operative exercise training programme for people with newly diagnosed cancers. Attendance at the cancer prepare sessions vary from eight to 12 people. The cost of the exercise programme was €7/8 (with/without a medical card) per session or €45/50 (with/without a medical card) per month (parking was included in this payment). Participants were encouraged to be physically active outside of the programme but no formal exercise advice or record log sheets were provided. The exercise intervention in this study was supervised by trained personnel with a background in cancer, and all MedEx staff are certified with the British Association for Cardiovascular Prevention and Rehabilitation and Basic Life Support.

The exercise prescription for interval (moderate to high intensity) exercise training and strength training is described using by the FITT-P (frequency, intensity, time, type, progression) principle (Table 1). The high-intensity exercise training was only prescribed to certain individuals who could tolerate the interval training very well. Exercise intensities were prescribed using the Rating of Perceived Exertion (RPE) scale. The RPE scale is a psychophysiological measurement that translates physical stimuli to a psychological construct of perceived exertion and has been validated in other clinical groups (Rosales et al. 2016). Adherence was assessed by the number of sessions attended, recorded by the lead researcher (LL).

**Table 1:** Exercise training prescription described using the FITT principle (frequency, intensity, time, type) and progression

Frequency	3–5 sessions per week depending on the time interval before surgery (i.e. if patients had a 2-week interval, they were advised to undertake 5 sessions per week or > 2 weeks, they were advised to undertake ≥ 3 sessions per week).
Intensity	(1) Interval (moderate to high intensity) exercise training: moderate and high intensities, derived based on RPE scale (13: somewhat hard and 15: hard) using the same concept as (West et al. 2014) without the use of CPET to inform intensities. (2) High-intensity exercise training: derived based on RPE scale (16: hard to very hard) using the same concept as (Boereboom et al. 2016) but without the use of CPET. (3) Resistance training included performing 3 sets × 12 repetitions. The load was selected based on individual ability (i.e. kg of weight using 12 RM with a minimum 30 s recovery period between each set)
Time	Total: ranged between 40 to 60 min “(1) The first interval (moderate to high intensity) exercise session was 30 min: 5-min warm-up followed by 4 repeated bouts of moderate intensity (3 min) to high-intensity (2 min) intervals and 5 min cool down. The second sessions onwards was 40 min: 5-min warm-up followed by 6 repeated bouts of moderate intensity (3 min) to high intensity (2 min) intervals and 5 min cool down. (2) The high-intensity exercise training was 17.5 min: 2 min warm-up followed by 5 repeated bouts of high intensity (1 min) and recovery (90 s) intervals and a 3 min cool down.” (3) Resistance training involved completing 3 sets × 12 repetitions (approx. 20 min).
Type	Aerobic training, upright cycle ergometer, recumbent cycle ergometer, treadmill, elliptical ergometer and rowing ergometer, depending on patient preference. Resistance training involved a circuit of strength 8–10 stations alternating upper and lower body exercises using the following machines: shoulder press, lateral pulldown, tricep press, squat, chest press, leg extension, hamstring curl, and back row.
Progression	Exercise intensity (interval/high) was progressed every 5 sessions (i.e. the intensity was increased by 1 level). Resistance training progressed following completion of 3 consecutive sessions comfortably (i.e. weight was increased by 1 kg).

The aerobic high-intensity training was only prescribed to certain individuals who could tolerate the interval training very well. The aerobic exercise training included interval and high-intensity training, which were alternated between every second sessions

*RPE* rate of perceived exertion, *CPET* cardiopulmonary exercise test, *RM* repetition maximum

### Outcome measurements

All outcome measures listed below, except for compliance and adherence, were assessed at baseline and post-exercise intervention (within 1 week prior to surgery) by the lead author (LL).

### Compliance and adherence

Compliance was calculated by number of participants completing pre- and post-intervention assessments. Adherence to the exercise programme was calculated by number of sessions completed divided by the number of sessions prescribed, i.e. if participants had a time window < 2 weeks of pre-operative exercise training, they were asked to exercise five times per week or if surgery > 2 weeks, they were asked to exercise three times per week.

### Effectiveness: HR components of fitness

#### Functional exercise capacity

Functional exercise capacity was assessed using the 6-min time trial (6MTT). Participants were instructed to cover as much ground as possible up and down a flat indoor 20 m course in 6 min by walking, running or a combination of both. Participants received a standard set of instructions and standard encouragement as per instructions used according to the ATS guidelines for the 6-min walk test (6MWD) (Holland et al. 2014). Total distance covered was recorded. A clinically meaningful improvement in 6MWD has been defined as an increase in distance by at least 19 m (Antonescu et al. 2014).

#### Strength

Hand grip strength was assessed on the dominant arm using a hydraulic handheld dynamometer (Takei 5401 digital dynamometer) which consisted of gripping a handle with a strain-gauge and an analogue reading scale. Participants were instructed to squeeze the gripping handle with maximum force for three seconds to provide a measure of isometric muscular strength of the hand and forearm. They were asked to repeat this three times with sufficient rest in between each effort and an average was recorded (Mathiowetz et al. 1984). A clinically meaningful improvement has been defined as an increase in grip strength ranging from 0.04 kg to 6.5 kg (Bohannon 2019). Lower body strength was assessed using the 10-repetition sit to stand test. Participants were asked to sit on a chair (height 44–47 cm) with their arms crossed on their chest, legs flat on the floor, parallel to each other and approximately shoulder width apart. They were asked to stand up and sit down 10 times as quickly as possible two times and the best time was recorded (Ozalevli et al. 2007). If participants were unable to complete the test, they were permitted to use their arms to assist however, all participants in this study were able to complete the test. A clinically meaningful improvement has been defined in sit-to-stand (for 5 repetitions) as 1.7 s in people with chronic lung obstruction (Jones et al. 2013), with limited literature on people with cancer.

#### Effectiveness: HRQoL

HRQoL was assessed using the European Organization for Research and Treatment of Cancer Quality of Life Questionnaire (EORTC QLQ-C30; 30 items) and EQ-5D. The EORTC QLQ-30 questionnaire is specific to cancer patients and is validated to assess generic aspects of HRQoL (Aaronson et al. 1993). A clinically meaningful change has been defined at least a 6-point decrease or at least a 3-point increase on QLQ-C30 domains (Hong et al. 2013). The EQ-5D is a simple descriptive profile and a single index value for health status (Rabin and de Charro 2001). Participants were encouraged to fill out the questionnaire independently but where assistance was required a member of their family, their friend or one of the research team filled out the questionnaire for the participant. A clinically meaningful improvement has been defined as 0.08 or 0.06 for UK- and US-based scores, and 0.07 for visual analogue scale (VAS) scores (Pickard et al. 2007).

#### Statistical methods

This was a pilot study therefore, no priori formal power calculation was undertaken. Data are presented for both cancer groups separately, and for the purpose of the pilot study, data are also reported for both surgical-oncology groups combined (prostate and CRC).

Continuous variables are reported as mean (range), mean (SD) or median (IQR), depending on distribution, and categorical variables as frequency (%). The Shapiro-Wilk test for normality of distributions was applied. For continuous variables, a paired *t* test was used to compare means between baseline and post-intervention for each surgical oncology group and for both surgical oncology groups, when relevant distributional assumptions were met and a Wilcoxin test otherwise. *P* < 0.05 was taken as statistically significant. All analyses were performed with the statistical software IBM SPSS Statistics Ver.23 (IBM Corporation, Armonk, NY, USA).

## Results

Thirty-two participants were recruited (15 prostate and 17 CRC). Baseline characteristics are shown in Table 2. Of the 32, 15 (47 %) had a co-morbidity including hypertension, hypercholesterolemia, type 2 diabetes, heart disease and arthritis, and 13 (41 %) were taking medication. Only one participant reported being a current smoker.

**Table 2:** Participant characteristics

	Prostate (n=15)	Colorectal (n=17)	P-value	Overall (n=32)
Gender M:F (ratio)	15:0	13:4	0.168	28:4
Age (years)	64.2 (6.7)	60.5 (12.1)	0.047*	60.5 (10.9)
Height (cm)	174 (5.7)	172 (8.5)	0.993	174.3 (5.9)
Weight (kg)	89.6 (12.5)	87.7 (21.6)	0.883	89.6 (16.4)
BMI (kg/m^2^)	29.7 (3.5)	29.5 (6.9)	0.898	29.7 (4.8)
Current smoker^┼^	0 (0)	1 (6)	0.343	1 (3)
No. of participants with co-morbidity^┼^	8 (53)	7 (6)	0.431	15 (47)
No. of participants taking medication^┼^	7 (47)	6 (35)	0.538	13 (41)

Data are reported as mean (SD).*P < 0.05 taken as statistically significant. ^┼^Frequencies with percentages in parentheses

Median (interquartile range (IQR) number of days between initial contact with the participant following referral at outpatient clinic (at point of cancer diagnosis) and baseline assessment at MedEx was 2 (1–4) days and 6 (2–8) days for the prostate cancer and CRC group, respectively. Fourteen of the 15 prostate cancer participants (93 %) completed the study (1 dropped out due to change in treatment pathway) whilst 10 of the 17 CRC participants (65 %) completed the study. Of these, 6 were unable to attend for follow-up assessment either due to advancement of the date of surgery (*n* = 3), work commitments (*n* = 1), holidays (*n* = 1) or a medical issue (*n* = 1) and 1 dropped out due to disease progression. Note: three of the CRC participants that completed the study were scheduled for neoadjuvant cancer treatment (combination of chemoradiotherapy/combination of short course radiotherapy and chemotherapy) in the first instance. Baseline assessment for these participants was taken immediately following completion of cancer treatment, and their exercise training was delivered in the same manner as the other participants in the time window available before surgery.

The median (IQR) duration of pre-operative exercise training was 4 (3-4) weeks and 2 (1–3) weeks for the prostate cancer and CRC participants. Adherence rates to the exercise programme for the prostate cancer participants was mean (SD) 89 (20) % and for the CRC group 81 (21) %. The median (IQR) number of exercise sessions attended was 10 (8–12) and 6 (4–11) for the prostate cancer and CRC groups, respectively.

For the prostate cancer group who completed the exercise programme, the mean (SD) distance from participants home to the MedEx centre was 18.8 (17.1) km whilst for CRC group the mean (SD) distance was 18 (16) km. All participants tolerated the exercise sessions, and there were no adverse events during the pre-operative exercise training programme.

Tables 3 and 4 illustrate selected HR components of fitness at baseline and post-intervention for the prostate and CRC group, and both surgical-oncology groups overall, respectively. From pre- to post-exercise intervention, there was a statistically significant improvement in lower body strength in the prostate cancer group mean difference (95% CI) 2.2 (0.6, 4.3) seconds, *p* = 0.045, and in the CRC group: 2.8 (1.5, 4.2) seconds, *p* = 0.001 and for both groups overall: 2.5 (1.2, 3.7) seconds, *p* = 0.001.

**Table 3:** Health-related components of fitness at baseline and post-exercise intervention for prostate and colorectal cancer participants

	Prostate (n=14)				Colorectal (n=10)			
Outcome measure	Baseline	Post-intervention	Mean Difference (95% CI)	P value	Baseline	Post-intervention	Mean Difference (95% CI)	P value
Sit-to-stand (s)	16.4 (6.6)	14.2 (6.1)	2.2 (0.6, 4.3)	0.045*	16.6 (5.9)	13.8 (4.9)	2.8 (1.5, 4.2)	0.001*
Handgrip (kg)	32.6 (9.6)	33.6 (8.6)	-1.0 (-2.8, 0.8)	0.259	33.7 (8.9)	35.3 (8.5)	-0.5 (-2.9, 1.9)	0.637
6MTT (m)	684 (144)	722 (136)	-38 (-108, 32)	0.262	768 (230)	779 (220)	-11 (-51, 28)	0.528

Data are presented as mean (SD). *P < 0.05 taken as statistically significant

**Table 4:** Health-related components of fitness at baseline and post-exercise intervention for both surgical-oncology groups combined

Outcome measure	Baseline (*n* = 24)	Post-intervention (*n* = 24)	Mean difference (95% CI)	*P* value
Sit-to-stand (s)	16.5 (6.2)	14.0 (5.5)	2.5 (1.2, 3.7)	0.001*
Handgrip (kg)	33.2 (9.1)	34.3 (8.4)	− 0.8 (− 2.1, 0.5)	0.232
6MTT (m)	719 (185)	746 (173)	− 27 (− 68, 15)	0.193

Data are presented as mean (SD). *P < 0.05 taken as statistically significant

Although no significant changes were reported for the upper body strength and 6MTT, there was a trend towards improvement from baseline to post-intervention in both surgical-oncology groups.

Tables 5 and 6 illustrates HRQoL at baseline and post-intervention for the prostate and CRC group, and for both surgical-oncology groups overall. For the EORTC QLQ-C30 questionnaire, there was a statistically significant improvement in global health status in the CRC group mean difference (95% CI) − 15.7 (− 28.7, − 2.8), *p* = 0.025 and for both groups overall − 10.6 (− 19.6, − 1.6), *p* = 0.023. There were similar improvements for emotional health subscale for the prostate cancer group mean difference (95% CI) − 10.9 (− 21.7, − 0.1), *p* = 0.048, and for both groups overall − 11.3 (− 21.2, − 1.4), *p* = 0.027. Additionally, there was a statistically significant improvement in nausea/vomiting/pain subscale for the CRC group mean difference (95% CI) 2.8 (1.2, 4.4), *p* = 0.005; and for both groups overall, 2.2 (0.2, 4.1), *p* = 0.030. For the EQ-5D questionnaire, there was a significant improvement in health scale status for the prostate cancer group mean difference (95% CI) − 8 (− 14.4, − 1.6), *p* = 0.019, and for both groups overall − 9.3 (− 15.6, − 3.1), *p* = 0.006.

**Table 5:** Health-related quality of life at baseline and post-exercise intervention for prostate and colorectal cancer participants

HRQoL Questionnaires	Prostate				Colorectal			
EORTC QLQ-C30 questionnaire^┼^	Baseline	Post-intervention	Mean Difference (95% CI)	P value	Baseline	Post-intervention	Mean Difference (95% CI)	P value
*Global health status*	73.1 (19.0)	80.1 (15.4)	-7.1 (-20.5, 6.4)	0.276	69.2 (15.2)	83.3 (13.6)	-15.7 (-28.7, -2.8)	0.025*
*Physical functioning*	93.8 (8.8)	92.8 (6.9)	1.0 (-2.2, 4.2)	0.505	94 (10.2)	94.7 (11.7)	-0.7 (-4.7, 3.3)	0.677
*Role functioning*	93.1 (19.4)	89.7 (19.9)	-1.4 (-16, 13.2)	0.838	90.7 (14.7)	92.6 (12.1)	-2.1 (-15.9, 11.7)	0.729
*Emotional functioning*	73.7 (19.2)	84.6 (13.1)	-10.9 (-21.7, -0.1)	0.048*	75.2 (28.6)	85 (10.2)	-11.8 (-34, 10.2)	0.293
*Cognitive functioning*	89.7 (14.5)	91.0 (11.0)	-1.3 (-10, 7.4)	0.754	93.3 (11.7)	100 (0)	-7.4 (-16.7, 1.9)	0.104
*Social functioning*	87.2 (26.5)	92.3 (12.9)	-5.1 (-18.4, 8.1)	0.416	86.7 (18.9)	90 (11.7)	-3.7 (-19.1, 11.7)	0.591
*Functional functioning*	97.7 (3.3)	97.7 (2.3)	-0.01 (-1.7, 1.7)	0.985	98 (2)	98.5 (1.2)	-0.6 (-2, 0.8)	0.359
*Fatigue*	18.5 (15.2)	13.7 (10.2)	5.6 (-3.2, 14.3)	0.191	28.6 (19.1)	33.3 (40.1)	-5.6 (-61.1, 50)	0.803
*Nausea/vomiting/pain*	5.2 (8.0)	3.2 (4.5)	1.7 (-1.7, 5.2)	0.294	5.8 (5.3)	3.3 (4.7)	2.8 (1.2, 4.4)	0.005***
EQ-5D questionnaire ^**ǂ**^								
*Mobility*				0.436				1.000
No problems	11 (85)	12 (92)			7 (78)	8 (89)		
Slight problems	1 (8)	1 (8)			1 (11)	0 (0)		
Moderate problems	1 (8)	0 (0)			1 (11)	0 (0)		
Severe problems	0 (0)	0 (0)			0 (0)	1 (11)		
*Usual Activities*								0.192
No problems	11 (85)	11 (85)		0.674	5 (56)	8 (89)		
Slight problems	0 (0)	1 (8)			4 (44)	1 (11)		
Moderate problems	2 (15)	1(8)			0 (0)	0 (0)		
*Self-care (washing/dressing)*								0.100
No problems	13 (100)	13 (100)			9 (100)	9 (100)		
*Pain / Discomfort*				0.191				1.000
None	11 (85)	7 (54)			6 (67)	7 (78)		
Slight	1 (8)	6 (46)			2 (22)	2 (22)		
Moderate	1 (8)	0 (0)			1 (11)	0 (0)		
*Anxiety / Depression*				0.586				0.676
None	6 (46)	6 (46)			6 (67)	7 (78)		
Slight	7 (54)	7 (54)			3 (33)	2 (22)		
*Health scale*	71.2 (16.4)	80.5 (14.5)	-8 (-14.4, -1.6)	0.019*	76.5 (15.1)	86.8 (6.4)	-11.1 (-25.1, 2.9)	0.104

Data are presented as mean (SD) and frequencies with percentages in parentheses. *P < 0.05 taken as statistically significant. ^┼^Prostate (*n* = 12), colorectal (*n* = 9); ^ǂ^prostate (*n* = 13), colorectal (*n* = 9)

**Table 6:** Health-related quality of life at baseline and post-exercise intervention for both surgical-oncology groups combined

EORTC QLQ-C30 questionnaire^┼^	Baseline	Post-intervention	Mean difference (95% CI)	*P* value
*Global health status*	71.4 (17.2)	81.5 (14.4)	− 10.6 (− 19.6, − 1.6)	0.023*
*Physical functioning*	93.9 (9.2)	93.6 (9.1)	0.3 (− 2, 2.6)	0.792
*Role functioning*	92.4 (16.8)	90.9 (16.8)	− 1.7 (− 11.1,7.8)	0.716
*Emotional functioning*	74.3 (23.2)	84.8 (11.7)	− 11.3 (− 21.2, − 1.4)	0.027*
*Cognitive functioning*	91.3 (13.2)	94.9 (9.3)	− 3.8 (− 9.8, 2.2)	0.204
*Social functioning*	87.0 (23)	91.3 (12.2)	− 4.5 (− 13.7,4.6)	0.315
*Functional functioning*	97.8 (2.8)	98.1 (1.9)	− 0.3 (− 1.3,0.8)	0.614
*Fatigue*	19.2 (17.6)	20.6 (25.8)	1.9 (− 13.7, 17.4)	0.805
*Nausea/vomiting/pain*	5.5 (6.7)	3.3 (4.5)	2.2 (0.2, 4.1)	0.030*
EQ-5D questionnaire^ǂ^				
*Mobility*				0.492
No problems	18 (82)	22 (100)		
Slight problems	2 (9)	0 (0)		
Moderate problems	2 (9)	0 (0)		
Severe problems	0 (0)	0 (0)		
*Usual Activities*				0.213
No problems	16 (73)	19 (86)		
Slight problems	4 (18)	2 (9)		
Moderate problems	2 (9)	1 (5)		
*Self-care (washing/dressing)*				
No problems	22 (100)	22 (100)		
*Pain/discomfort*				0.266
None	17 (77)	14 (64)		
Slight	3 (14)	8 (36)		
Moderate	2 (9)	0 (4)		
*Anxiety/depression*				0.492
None	12 (55)	13 (59)		
Slight	10 (45)	9 (41)		
*Health scale*	73.5 (15.7)	83.4 (11.7)	− 9.3 (− 15.6, − 3.1)	0.006*

Data are presented as mean (SD) and frequencies with percentages in parentheses.*P < 0.05 taken as statistically significant. ^┼^Overall (*n* = 21); ^ǂ^Overall (*n* = 22)

## Discussion

This study demonstrated that people with newly diagnosed prostate cancer and CRC facing major invasive surgery, who consented to participate, were amenable to undertaking a community-based pre-operative exercise programme, and were compliant to the exercise programme and the outcome assessments at follow-up. Additionally, the delivery of the programme, using a pragmatic approach, over a relatively short time window had high adherence rates, without any adverse events. The programme had significant effects on HR components of fitness and HRQoL in both surgical-oncology groups.

Pre-operative clinical guidelines and recommendations on exercise training have been recently published that provide practical guidance for providing safe and effective exercise (Tew et al. 2018). One of the guidelines recommends that exercise training should commence as early in the surgical pathway as possible ensuring a minimum training duration of 4 weeks. In the current study, participants commenced the exercise programme within a median (IQR) of 2 (1–4) days and 6 (2–8) days from the time of referral. Additionally, the pre-operative exercise training programme was delivered over a median (IQR) of 4 (3-4) weeks for the prostate cancer group and 2 (1–3) weeks for the CRC participants, in line with the standard local hospital waiting time. The duration of the exercise programme in the current study is similar to other previous pre-operative exercise studies (Mujovic et al. 2014; West et al. 2014; Barakat et al. 2014; Coats et al. 2013; Gillis et al. 2014). However, to our knowledge, the time between referral and commencement of exercise training in this context has not been documented. Our study shows that early access can be facilitated using a referral pathway from the local hospital to the community.

The delivery of the pre-operative exercise programmes vary but have predominantly been hospital-based with some home-based (Mujovic et al. 2014; West et al. 2014; Barakat et al. 2014; Coats et al. 2013; Gillis et al. 2014). Other exercise studies have previously investigated community-based pre-operative exercise training programmes (as a standalone component of prehabilitation) in the surgical-oncology setting (Rao et al. 2012; Singh et al. 2017). One breast cancer study included an exercise intervention (boot camp) during neoadjuvant cancer treatment prior to surgery (*n* = 10) and reported a compliance and adherence rate of 100% and > 80%, respectively (Rao et al. 2012). The exercise sessions however were delivered using a one-to-one supervised approach. Another prostate cancer study included exercise programme, delivered in an exercise facility at a university, and reported 100% compliance with moderate adherence with only half of the participants completing more than 80% of the sessions (Singh et al. 2017). In our study, 75% completed the study with an adherence rate of > 80%. Our compliance rates are somewhat lower than previous similar studies (however their sample sizes are lower) (Rao et al. 2012; Singh et al. 2017). Possible explanations for the high compliance rates for the prostate cancer group in the current study may due to participants starting the MedEx programme with a confirmed date of surgery whilst many of the CRC group started the programme with no date confirmed. It is important to highlight that the CRC pathway is more complex as patients who present with a risk of obstruction may require surgery immediately. The adherence rates of > 80 % in the current study are encouraging and appear to be higher than previous home-based programmes which vary widely between 16% (Carli et al. 2010) and 74% (Kim et al. 2009) in CRC studies. In contrast, however, they are similar to hospital-based programmes which have been reported to be > 80% (West et al. 2014; Morielli et al. 2016) in CRC studies, 70–100 % in an abdominal aortic aneurysm study (Kothmann et al. 2009) and 81% in a lung cancer study (Mujovic et al. 2014). The high adherence rates in the current study may be due to the consultant surgeon referring the patient to the programme and the nature of the MedEx programme being medically supervised. Additionally, it may be due to the exercise group being delivered a part of the MedEx exercise programme (group-based). The high adherence rates in the prostate cancer group in our study may be important as a previous study reported that men scheduled for radical prostatectomy had poor adherence to healthy lifestyle recommendations from the World Cancer Research Fund (WCRF) and the American Institute for Cancer Research (AICF). Sixty-seven percent did not fulfil the criteria of a normal healthy weight, 33.5 % reported doing no exercise and 49.6 % were current or ex-smokers (Thederan et al. 2019). Community-based interventions could potentially play an integral role in promoting such recommendations prior to prostatectomy.

Participation in the exercise programme had a positive improvement in lower body strength in both surgical-oncology groups. We also showed improvements in upper body strength and functional exercise capacity, although changes were not statistically significant. Improvements in HR components of fitness may be of importance; however, currently, the evidence base pre-operatively in this area has focused on cardiopulmonary exercise testing (CPET) variables in hospital settings. Participation in the community-based pre-operative exercise programme had a significant improvement on certain domains of HRQoL in both surgical-oncology groups. These findings may be important as psychological variables appear to be associated with early surgical recovery (Mavros et al. 2011). One community-based programme in people with breast cancer reported significant improvements in body mass index and Ki-67 levels (i.e. prognostic and predictive marker of cancer diagnosis) following investigating the effects of a boot camp intervention (Rao et al. 2012). Another study in prostate cancer reported significant improvements in muscle strength and physical performance (6-m fast walk, 400-m walk and repeated chair rise test) following a pre-operative community-based programme which was delivered in the university exercise clinic (Singh et al. 2017). Our findings show that the community-based model is effective in improving HR components and HRQoL.

The recently published pre-operative exercise training guidelines (Tew et al. 2018) state that if resources are limited, priority of pre-operative exercise training referral should go to patients who are at increased risk of peri-operative complications. This community-based model allows for greater accessibility pre-operatively and has the potential to make the programme available to all eligible patients and not just those who are at increased risk. A previous community-based study in people with prostate cancer reported that four out of 14 eligible patients refused to participate in the study citing travelling from their residence to the university exercise clinic where training and assessments took place as a barrier to participation (Singh et al. 2017). A recent qualitative study highlighted that future exercise programmes should include a mixture of supervised group sessions within a hospital- or community-based centre and advice about exercise at home (Crandall et al. 2019). Future community-based programmes, which includes both centre- and home-based exercise programmes, may accommodate patient preferences and circumstances, hence removing barriers to participation. The MedEx model is a sustainable alternative to hospital-based programmes, reducing significant healthcare costs and providing easier access to patients (with the addition of new centres being established nationally). Future work should include health economic analyses to investigate cost savings in this regard.

Strengths of this work include the novelty of the community-based setting. Additionally, the rapid referral and access from the local hospital to the community. However, the study has several limitations. Due to a lack of funding to support this research, there were no resources available to cover personnel costs. Therefore, no study uptake rates (screening logs) were recorded and thus we are unable to report on the number of patients approached versus number of patients who agreed to participate. Note: the cancer prepare service at ExWell (formerly MedEx) now runs daily from Mon-Fri which highlights the feasibility of this exercise-oncology service. Participants in this study had little comorbidity with a reasonable level of functioning; therefore, it is possible they were a motivated group which increases the risk of selection bias (impacting both internal and external validity). Due to the nature of the pilot study, no usual care control group were included. Functional exercise capacity was measured using a field-based measure; howeve CPETr was not possible in the community setting. Three CRC participants completed neoadjuvant cancer treatment in the first instance whilst the remaining were scheduled for surgery only; therefore, heterogeneity exists in this cancer group. Furthermore, data on surgical intervention and patient clinical outcome was not provided as hospital ethics were not obtained. The sample population in the CRC group largely consisted of males (87%) which may be a potential bias as CRC incidence rates in Ireland is 42% for females and 58% for males. Additionally, the mean (SD) age of the CRC group in the current study is 60.5 (12.1) years. However, approximately half of people diagnosed with CRC in Ireland are aged 70 and older (51% of men and 58% of women); therefore, the sample may not be representative and limits the generalizability of our findings.

## Conclusion

People with newly diagnosed prostate and CRC in our study facing major invasive surgery were amenable to undertaking a pre-operative exercise programme in the community. Additionally, the programme had an acceptable compliance rate and a high adherence rate, and significantly improved HR components of fitness and HRQoL. Future adequately powered trials are warranted to investigate the effects of a community-based programmes on important patient reported outcomes and clinical outcomes.

## Data Availability

The datasets used and/or analysed during the current study are available from the corresponding author on reasonable request.

## Abbreviations

RPE: Rate of perceived exertion
CPET: Cardiopulmonary exercise test
RM: Repetition maximum
